# Analysing self-regulatory behaviours in response to daily weighing: a think-aloud study with follow-up interviews

**DOI:** 10.1080/08870446.2019.1626394

**Published:** 2019-06-14

**Authors:** Kerstin Frie, Jamie Hartmann-Boyce, Caitlin Pilbeam, Susan Jebb, Paul Aveyard

**Affiliations:** Nuffield Department of Primary Care Health Sciences, University of Oxford, Oxford, United Kingdom

**Keywords:** Self-regulation, self-monitoring, daily weighing, weight loss, action planning

## Abstract

**Objective:** To examine the extent to which people who are trying to lose weight naturally self-regulate in response to self-weighing and to identify barriers to self-regulation. **Design/Main Outcome Measures:** Twenty-four participants, who were overweight and trying to lose weight, recorded their thoughts during daily weighing for eight weeks. Semi-structured follow-up interviews assessed participant experiences. Qualitative analysis identified steps of the self-regulation process and barriers to self-regulation. Exploratory regression analysis assessed the relationship between the self-regulation steps and weight loss. **Results:** On 90% of 498 occasions, participants compared their weight measurement to an expectation or goal, and on 58% they reflected on previous behaviour. Action planning only occurred on 20% of occasions, and specific action planning was rare (6%). Only specific action planning significantly predicted weight loss (−2.1 kg per 1 SD increase in the predictor, 95% CI = −3.9, −0.3). Thematic analysis revealed that barriers to the interpretation of daily weight changes were difficulties in understanding day-to-day fluctuations, losing the overview of trends, forgetting to weigh, and forgetting previous measurements. **Conclusion:** Specific action planning can lead to weight loss, but is rare in a naturalistic setting. Barriers to self-regulation relate to the interpretation of weight changes.

## Introduction

Self-monitoring of weight is one of the most commonly used behaviour change techniques in weight management interventions (Burke, Wang, & Sevick, 2011), and recent studies indicate that it has a beneficial effect for weight loss (Helander, Vuorinen, Wansink, & Korhonen, 2014; LaRose, Gorin, & Wing, 2009; Shieh, Knisely, Clark, & Carpenter, 2016; Steinberg et al., 2013). A meta-analysis found that self-monitoring of weight, when embedded in a weight loss programme, significantly increased weight loss by 1.7 kg (Madigan, Daley, Lewis, Aveyard, & Jolly, 2015). Observational data suggests the frequency of weighing is positively associated with weight loss (Helander et al., 2014; LaRose, Lanoye, Tate, & Wing, 2016; Steinberg, Bennett, Askew, & Tate, 2015). Several reviews have found evidence that weighing does not lead to adverse psychological consequences, including negative affect, body-related attitudes or disordered eating (Madigan et al., 2015; Shieh et al., 2016; Zheng et al., 2015) (Benn, Webb, Chang, & Harkin, 2016).

The effectiveness of self-monitoring is hypothesised to be based on a self-regulation process, whereby monitoring oneself allows for (1) the comparison of the current status to a previously set goal, thus providing (2) the opportunity to reflect on the effectiveness of previous behaviour, and enabling (3) the formulation of an action plan to reach the goal, which is followed by (4) the performance of the planned action (Boutelle, 2006; Kanfer & Karoly, 1972; LaRose et al., 2009). Previous research has extensively studied the connection of steps (3) and (4) of the self-regulation process, especially focussing on the intention-behaviour gap (Adriaanse, Vinkers, De Ridder, Hox, & De Wit, 2011; Sheeran, 2002; Sheeran and Webb, 2016). This research has shown that intentions do not predict behaviour as well as they should in theory, because people often do not follow through with their action plans (Sheeran, 2002). However, to the best of our knowledge, there has been little research looking at the hypothesised predecessors of intentions and behaviours in the self-regulation process, that is, the more cognitive and therefore hidden processes ‘comparison to goal’ and ‘reflection’, as well as their connection to ‘action-planning’. It is unclear whether people perform these processes as part of self-regulation. It is also unknown to what extent this cognitive part of the self-regulation process occurs naturally after weighing. That is, it is currently unclear whether self-regulation is automatically triggered upon weighing, or whether further weight loss treatment components are necessary to support people to engage in this process, as studies of self-weighing have mostly incorporated considerable additional behavioural support.

The purpose of this study is therefore to examine the extent to which the cognitive aspects of self-regulation occur unpromptedly during daily weighing in people who are overweight and trying to lose weight. To this end, this study investigates the thoughts and feelings people have during and after daily weighing. We further explore the relationship between self-regulation and weight loss outcomes after eight weeks, to investigate which aspects of the self-regulation process may be driving the weight loss effect found to be associated with self-monitoring. The study additionally aims to explore participant experiences with daily weighing to identify in-the-moment barriers to self-regulation and daily weighing.

## Methods

### Participants

For the sample size estimation, we were guided by information power considerations, as described by Malterud, Siersma, and Guassora (2016). These considerations included the aim of the research, the usage of theory, the specificity of the sample, the quality of data, and the focus of analysis. We considered that this primarily qualitative study had a clearly defined aim, used existing theory, and would sample participants purposefully. Since we could not predict the quality of the data, we decided to acquire a large data set from each participant, comprising daily measures over eight weeks. While giving us enough data for the qualitative analysis, this length of the study also allowed us to analyse weight loss effects of self-regulation in our exploratory analysis, as previous studies have been able to detect weight loss effects after 2 months (Lally, Chipperfield, & Wardle, 2008). Cross-case comparison in the analysis meant that the sample size had to be large. Following these considerations, a sample size of 24 was deemed sufficient. Participants were recruited through social media and advertisements in public places from May to July, 2017. Criteria for inclusion were a body mass index of ≥25 kg/m^2^, the aim to lose weight, sufficient English language skills to ensure that content and depth of think-aloud recordings would not be biased by language difficulties, and age ≥18 years. Participants were excluded from the study if they already weighed themselves more than once a week, in order to recruit participants for whom daily weighing would be a new behaviour. Participants were also excluded if they reported having lost more than 5% of their current body weight in the previous six months to ensure participants were not already following a successful weight loss routine which might influence their engagement with the weight measurements. Participants were excluded if they were pregnant or planning a pregnancy, if they were taking medication associated with weight gain, or if they had ever had bariatric surgery as all of these would affect the weight measurements. Participants were excluded if they had a history of eating disorders as daily weighing might cause adverse effects in this group of people.

### Procedure

Participants were invited to two meetings, one at baseline (∼45 min) and another in the 9th week of the study (∼60 min). At both sessions, height, weight, and body fat were measured objectively by a researcher. In the first session, participants were briefed on the study tasks. Participants were asked to document their thoughts during weighing every day for eight weeks. They were provided with scales equipped with a SIM card (BodyTrace) to automatically synchronize recorded weight measurements with a secure research server via the 3G/4G network. To reduce measurement bias, participants were asked to place the scales on a hard surface and weigh themselves first thing in the morning. Participants were asked to use the think aloud method (Van Someren, Barnard, & Sandberg, 1994). They were instructed to verbalise all thoughts that came to their mind during and right after the weighing process and not to filter the information shared. They were told not to create content if they had nothing to say. They were asked to mention the date and weight measurement on the recording. They received a demonstration of how to use the scales and how to make audio recordings on a smartphone. Participants were asked to fill in a short online questionnaire on their previous experiences with weighing and their expectations concerning the study. Finally, participants were encouraged to send their first think-aloud recording to the research team to receive feedback on the quality of the recording and the completeness of information (i.e. mentioning of date and weight measurement). After 4 weeks of daily weighing, participants were sent an online questionnaire asking about their first experiences with self-weighing in the study. At the second university-based session, all participants were interviewed using a semi-structured interview, debriefed, thanked for their contribution and given a £25 honorarium.

### Measures

#### Height and weight measurements

Participants height, weight, and body fat was measured at baseline and follow-up using a freestanding stadiometer (Tanita Corporation, Leicester Height Measure MkII) and a body composition analyser working with bioelectrical impedance (Tanita Corporation, BC-418MA body composition analyser).

#### Think-aloud recordings

Participants recorded their daily thoughts during weighing over eight weeks. Participants could choose for themselves whether they preferred to think aloud in audio-recordings or in written diary entries and could switch methods throughout the study. The major advantage of this method was that we were able to capture participants’ thoughts in the moment of their occurrence, and in a natural environment without external prompting. Participants received text messages every other day to remind them of their daily weigh-in. We called participants every two weeks to ask whether they experienced any problems. Participants were asked to submit their daily think-aloud recordings by the end of the study. Resources did not allow us to transcribe recordings from all weeks. We therefore had to restrict our qualitative analysis to data from three weeks of the study. We decided to exclude the first week as we found that participants mostly commented on the functioning of the scales and the procedure. The 8th week was also excluded as many comments revolved around the upcoming end of the study. In order to consider data from different stages of the study, the 2nd, 4th, and 7th week of think-aloud recordings were transcribed and analysed. Where think-aloud recordings were missing, the next available day’s recording was extracted instead until three complete weeks were included in the analysis. This strategy was successful for 23 participants; for the 24th participant the last two weeks of data were missing. Thus, 21 data points were analysed for 23 participants, and 15 data points for the 24th participant.

#### Semi-structured interview

In the semi-structured interview at the end of the second meeting, we explored participants’ experiences with self-weighing. We conducted these interviews as we wanted to ensure that we would be able to capture any significant themes regarding participant experiences and barriers, in case the think-aloud recordings would not cover this information. In addition, the semi-structured interviews allowed us to put the think-aloud recordings into context and collect some information on the weight loss approaches used by the participants.

One researcher conducted all interviews and was trained in qualitative interviewing by a senior qualitative researcher before meeting participants. We asked participants how they felt about self-weighing and which aspects they liked and disliked, whether they tracked their weight measurements throughout the eight weeks, whether they thought weighing was useful for controlling weight, which methods they used to try to lose weight, and their intentions to continue weighing. Follow-up questions were asked in response to statements made by the participants, thus allowing flexibility of topic coverage. As participants varied in the detail of their answers, the interviews lasted between 20 to 60 minutes. All interviews were transcribed and analysed.

### Data analysis

Adherence rates were calculated for each participant and correlated with weight change to assess whether the frequency of task performance influenced the outcome. Adherence was defined as the presence of both a weight record and a think-aloud recording for any given day.

The content of the think-aloud recordings was analysed using a mixed-methods approach. In order to analyse the extent to which self-regulation processes occurred during weighing, we employed a combination of framework (Ritchie & Spencer, 2002) and content analysis (Silverman, 2014), using the software NVivo (QSR International, Version 11.4.2). To this end, we created an *a priori* framework, including (1) the comparison to an expectation or goal, (2) the reflection on previous behaviour, (3) general action planning, not defining a specific action or time plan (e.g. ‘I will do some exercise today’), and (4) specific action planning, i.e., defining a concrete action and time plan (e.g. ‘I will go for a run during my lunch break today’). Action planning was split into two categories, as the literature suggests that concrete action plans are more likely to be implemented than vague ones (de Vet, Oenema, & Brug, 2011). After familiarisation with the data, coding was performed by two independent researchers for 25% of the participants, reaching good reliability scores (mean κ = 0.97). The rest of the think-aloud recordings were coded and analysed by one researcher. Coding was quantified as proportions by calculating per person average occurrences of each self-regulation step over the 21 days analysed (see Supplemental Material 1). Using the statistical software package SPSS (IBM, Version 24), we examined the associations between the self-regulation steps with Pearson correlations. Exploratory regression analysis assessed the relationship between self-regulation scores and weight loss success.

Additionally, themes related to experiences with daily weighing and self-regulation were identified in the think-aloud recordings using inductive thematic analysis (Braun & Clarke, 2012). Following familiarisation with the data, themes were generated from recurring topics and, where possible, using participants’ own words to preserve closeness to the data. No a priori framework or ideas were imposed onto the data and we only recorded themes that emerged from the recordings. Coding was performed by two independent researchers for six participants, reaching fair to good reliability scores (mean κ = 0.93). The rest of the think-aloud recordings were coded and analysed by one researcher. Codings were matched to overarching themes using an OSOP (‘One Sheet Of Paper’) analysis (Ziebland & McPherson, 2006). We aggregated codes on one piece of paper and established links between them, which enabled us to identify overarching themes.

As we found the analysis of the think-aloud recordings to be very fruitful, we took a deductive approach with the semi-structured interviews to validate the themes that emerged from the thematic analysis of the think-aloud interviews. Statements exemplifying identified themes from the thematic analysis of the think-aloud recordings were extracted. In addition, details concerning the participants’ weight-tracking behaviour, perception of usefulness of daily weighing, and intentions to continue weighing were extracted from the semi-structured interviews and quantified as counts (see Supplemental Material 1).

## Results

### Sample characteristics

Of the 94 people who expressed their interest in the study, 83 were reachable by phone and interviewed for eligibility, and 25 were eligible to participate. Twenty-four participants completed the full eight weeks of the study. One male participant was lost to follow-up for unknown reasons and his data was therefore not available for analysis. The final sample consisted of 9 male and 15 female participants; mean age 36.6 years (SD = 13.27), mean weight at baseline 85.0 kg (SD = 17.9 kg), mean body mass index (BMI) at baseline 29.60 (SD = 4.76). Twenty-two participants had a university degree or equivalent, ethnicity was mixed. Eight participants were students. Seven participants weighed themselves once a week before the study began, all others weighed themselves less often. For full details on sample characteristics, see Table 1. Seventeen participants chose to audio-record their thoughts, two wrote them into a journal, and six used a mixture of both methods.

**Table 1.** Demographic characteristics of the sample.

Characteristics		*N* (out of 24)
Gender	Male	9
	Female	15
Ethnicity	White-British	13
	White-Other	5
	Asian	4
	Mixed	2
Highest educational qualification	Degree or equivalent	22
	A’ levels, vocational level 3 and above	2
Employment status	Employed or self-employed	16
	Looking after home/family	2
	Student	8
Weighing Frequency Before Study Begin	Less than once a month	7
	Once a month	6
	Once every other week	4
	Once a week	7
Liking of Weighing Before Study Begin	Dislike it a great deal	3
	Dislike it somewhat	10
	Neither like nor dislike it	8
	Like it somewhat	3
**Characteristics**	**Mean**	**SD**
Age	36.6 years	13.27 years
Weight at baseline	85.0 kg	17.9 kg
BMI at baseline	29.60	4.76

### Adherence and weight loss outcome

Participants weighed themselves and thought aloud on 93% of the 56 study days. Average weight change after eight weeks was −0.89 kg (SD = 3.30). This included one participant who lost 13.6 kg. Excluding this outlier, there was a small average change in weight of −0.33 kg (SD = 1.93 kg). A Pearson correlation showed that adherence to daily weighing and think-aloud recording was not related to weight change (*r*= −0.059, *p* = 0.785), this remained unchanged when excluding the outlier (*r* = 0.082, *p* = 0.711).

### Self-regulation occurrence and correlations

The framework and content analysis revealed considerable differences in the occurrence rates of the different self-regulation steps (see Table 2). On 90% out of 498 of occasions, participants compared their current weight to an expectation or goal weight. On slightly more than half (58%) of occasions, participants reflected on previous behaviours that contributed to the weight change observed. However, on only 20% of occasions, participants performed any action planning. Specific action planning was rare (6%). Participants who frequently performed the comparison step were more likely to reflect on previous behaviours (*r* = 0.678, *p* < 0.001). Participants who performed the reflection step were more likely to make general action plans (*r* = 0.446, *p* = 0.029). There was a marginally significant effect for participants who reflected to make more specific action plans (*r* = 0.390, *p* = 0.060). See Table 3 for all correlation results.

**Table 2.** Frequency of occurrence of the different self-regulation steps in the think-aloud recordings.

Self-regulation step	Number of occasions self-regulation step was performed (out of 498 total occasions)	Number of participants performing self-regulation step at least once
1. Comparison	450 (90%)	24
2. Reflection	287 (58%)	24
3. General action planning	71 (14%)	16
4. Specific action planning	30 (6%)	12

**Table 3.** Correlational analysis investigating the associations of the self-regulation steps.

		Average comparison	Average reflection	Average general action planning	Average specific action planning
Average comparison	*r* (*p*)	1 (0)	0.678 (0.000)	0.365 (0.079)	−0.111 (0.605)
				
Average reflection	*r* (*p*)		1 (0)	0.446 (0.029)	0.390 (0.060)
				
Average general Action planning	*r* (*p*)			1 (0)	0.291 (0.167)
				
Average specific Action planning	*r* (*p*)				1 (0)
				

### Relationship of self-regulation and weight loss

The overall model testing the relationship between the self-regulation steps and weight loss in the complete sample was not significant (*F*(4, 19) = 1.905, *p* = 0.151). However, Cohen’s effect size *f*^2^ of the model revealed a large effect for the self-regulation steps (*f*^2^= 0.406). Post-hoc power analysis using G*Power (Version 3.1) revealed a power of 0.58 for the model. Only the specific action planning variable significantly negatively predicted weight change (−2.1 kg per 1 SD increase in specific action planning, 95% CI −3.9 to −0.3 kg, *p* = 0.027). When excluding the outlier, the relationship between the self-regulation steps and weight change was statistically significant (*F*(4, 18) = 5.762, *p* = 0.004). The effect size for the model further increased (*f*^2^= 1.282), and power analysis showed a power of 0.98. Specific action planning was the only individual component of self-regulation that predicted weight loss significantly (−1.4 kg per 1 SD increase in specific action planning, 95% CI −2.3 to −0.6 kg, *p* = 0.003). See Table 4 for all results.

**Table 4.** Linear regression predicting weight change from all self-regulation steps; with and without outlier.

			Predictor variables			
	Overall model significance	Statistic	Average comparison	Average reflection	Average general action planning	Average specific action planning
With outlier (*N* = 24)	*F*(4)=1.905	*B*^a^	−1.507	0.741	0.463	−2.057
	*p* = 0.151	*p*	0.175	0.521	0.539	0.027
	*f*^2^=0.406	CI^a^	−3.745; 0.731	−1.628; 3.110	−1.084; 2.010	−3.854;
	Power = 0.582					−0.261
Without outlier (*N* = 23)	*F*(4)=5.762	*B*^a^	−0.531	0.871	−0.678	−1.424
	*p* = 0.004	*p*	0.308	0.113	0.081	0.003
	*f*^2^=1.282	CI^a^	−1.594; 0.532	−0.228; 1.970	−1.449; 0.094	−2.286;
	Power = 0.980					−0.563

a The coefficients represent the effect of a 1 SD increase in the independent variable and show the change in weight in kg that might be expected to ensue.

### Experiences with and barriers to daily weighing and self-regulation

The most prominent themes from the thematic analysis are discussed in the following sections, complemented by quotes and quantified information extracted from the follow-up interviews. Saturation of data was reached as no new themes were generated from the analysis of the last six participants’ data.

### Weighing evokes emotional reactions

Self-weighing evoked an emotional response in participants. Reactions both within and between individuals comprised positive and negative responses, including relief, joy, shame, frustration, and guilt. The nature of the reaction depended on whether the weight outcome was higher or lower than expected. Emotional reactions were elicited in both men and women, although women tended to show a stronger emotional response.

It's definitely higher than what I expected [um] which makes me feel kind of anxious [um] and uncomfortable. (female)

I mean obviously I'm cheerful. There is nothing like seeing unexpectedly low numbers on the scale for making a person feel exceedingly jolly. (female)

Some female participants stated that they found their emotional response to the weight outcome more intense than they felt was appropriate.

And it is also absurd that my happiness can be so profoundly altered by seeing a number on the scale that's slightly, slightly less than I was expecting, and that – let's face it – is 40 kilos more than it should be. (female)

Positive weight changes encouraged participants in their weight loss attempt, while disappointing and frustrating weight measurements led some participants to want to give up on their weight loss attempt since they felt that their efforts were not fruitful anyway. In the debrief interview, one participant noted:

I think I get too upset when it doesn’t go down. When I’ve done a really good day of eating healthily and then I don’t see any difference on the scales […] I’m like oh what was the point. (female)

Some participants, both male and female, commented on becoming accustomed to the daily weighing routine. They found that their emotional reactions to the weight measurements reduced over time.

[I’m] feeling fewer strong emotions in either direction when getting on the scale. (male)

Further support for a habituation process was found in follow-up interviews, where nine participants commented on having noticed that their emotional reactions decreased in intensity throughout the study.

I think I feel better about weighing myself now[…], because you do it so often it’s not like a scary number anymore you kind of know what to expect and I think yes it’s less kind of horrible. (female)

One female participant started the study strongly disliking weighing herself and found that by the end she had developed more positive feelings towards it. In one of her think-aloud recordings she reported:

I'm finding I'm vaguely looking forward to weighing every day. Which is quite a big change about how I feel about the scale. (female)

She expressed worry about having to return the scales we lent her by the end of the study, which shows that she had found value in this part of her daily routine.

### Motivation and influence on daily life

Seeing the daily weight measurement prompted both male and female participants to think about changing their behaviours. This was often paired with statements expressing motivation, but an action plan was rarely made.

Boy oh boy, seven weeks and nothing to show for it. […] But it is good, it is giving me some very strong feedback. I can't fool myself any longer. This must change now. (male)

At the follow-up interviews, participants commented that daily weighing over eight weeks helped them realise that their approach to weight loss needed to change in order to lose weight.

It made me realise that I was not only in denial about how quickly I should be losing weight with what I’m doing, but actually probably in denial about what level of effort I’m actually making to lose that weight. (female)

Even though participants rarely made action plans in the think-aloud recordings, some noticed while reflecting on past behaviour that the daily weighing had affected their actions throughout the day.

And in fact, yesterday I was at a party in the afternoon and there was quite a lot of cake, I didn't eat any of it […]. And I think that was because of the awareness that I would be weighing. (female)

It [the weighing] is just like lightly in my head during the day and it is affecting, all be it very subtly, decisions that I'm making about what I'm eating and what I'm not eating. (female)

This effect of regular weighing acting as a reminder of the weight loss goal throughout the day was also mentioned by participants in the follow-up interviews.

But, I think the weighing was really mutually supportive. […] At least on a couple of days, I think it really did help me make the right choice in a moment where I might not have made the right choice otherwise, because I knew I was going to read that scale the next day. (male)

Hence, even without action planning the weighing process had influenced participants’ behaviour. This might also explain why 22 participants stated in the interviews that they found weighing useful for controlling their weight.

### Barriers to interpreting weight measurements

Participants struggled to make sense of their weight changes on a day-to-day basis. They reported in their think-aloud recordings that the daily fluctuations were not always clearly associated with their previous behaviour, making it hard to explain weight changes. This led to frustration as expectations were often not met. This was especially pronounced for female participants, male participants seemed to have fewer unexplainable weight changes and reacted less strongly to them.

My experience in the past is that if you've dieted and had what you think is a very good week about eating and it's not reflected on the scale, very often that can make you miserable and drive you into giving up, or relinquishing a bit of control, or just throwing the towel in because well, you felt deprivation and you're not getting the reward of the scale number. (female)

Participants were aware that the daily fluctuations could not only stem from fat loss but also from other changes in their body. Understanding the cause of a weight change was therefore perceived as difficult.

If there is one major thing that is stressing me out about my weight it’s that I feel I have no idea if the fluctuations I’m seeing are related to water consumption, fat or muscle loss, amount of food consumed, time I’m weighing myself. (female)

Confusion surrounding the interpretation of the weight changes and the meaning of fluctuations was sometimes followed by feelings of helplessness.

I'm not sure what I'm doing wrong, I'm trying to eat healthy, I'm doing exercise classes but the weight just isn't moving. I'm trying all sorts of different things. (female)

In the previous theme on emotions we identified that negative emotional reactions sometimes led participants to want to give up on their weight loss attempt. In the context of this theme, we see that negative emotional reactions were especially a barrier when participants could not explain what they had done wrong or what they should do differently.

Problems with interpreting daily weight changes were further aggravated by unrealistically high expectations about the rate of weight loss. For instance, the following participant expressed her frustration about a lack of weight loss although she was 400 g lighter than the previous day:

Still no move, still no change, still not making any difference to everything that I'm doing. Beyond frustrated. (female)

Similarly, a participant stated that she realised she had exaggerated expectations about weight changes in the follow-up interview:

Yes if I eat a pizza one day I’d be expecting it to be like 10 kilos more, which is, I know silly. (female)

Given that daily weight changes were difficult to interpret, participants tried to identify trends in their data. However, they reported difficulties in taking this longer-term view.

I guess one of the irritating things about weighing oneself daily is that improvement/weight loss is harder to spot. (female)

This mostly had to do with problems with keeping an overview of all measurements taken over time, as participants forgot previous weighing results.

If you weigh yourself every week there’s an obvious change and you can remember what you were the week before[…]. When you’re doing it daily you lose track of what your weight was the day before. (female)

Although 19 participants stated in the follow-up interview that weight-tracking might have facilitated identifying trends in the data, only 11 of the participants actually kept track of their weight throughout the study.

I kind of regretted […] not doing that [weight-tracking] as well because […] I have no idea actually what I weighed at the beginning of the study, […] maybe knowing what like each week sort of my progress had been if I was trying, that would have been a better like behavioural mechanism that could have prompted me to certain behaviours. (female)

Interpretation of the weight measurements was not helped by the fact that participants sometimes forgot to weigh themselves first thing in the morning, increasing the apparent daily fluctuations. Participants were aware of this problem and preferred weighing themselves in the morning.

I don’t like weighing myself last thing at night, want to start first thing again. Maybe I won’t stress about it so much then. (female)

Overall, continuing problems with the interpretation of weight changes emerged as a barrier to daily weighing and self-regulation.

### Weighing frequency

Twenty participants stated in the follow-up interviews that daily weighing had become a habit, which they had incorporated in their morning routine. All participants stated they would like to continue weighing themselves regularly, including eleven participants who stated they would like to continue weighing themselves daily. They found that daily weighing helped them to keep on track with their weight loss goals.

I guess at least now like every day I'm getting the push like you should do something different rather than like every week getting the push you should do something different. (female)

However, with daily weighing, participants were more likely to dismiss changes they did not want to see as being part of the natural fluctuations in weight.

It would kind of creep up quite easily, because each day I could just dismiss it […]. So I was kind of, it was easy to convince myself with oh no that’s just, you know, fluctuations. (female)

Based on the problems with interpreting daily weight changes, half of the participants specifically stated in the follow-up interviews that they would prefer less regular weighing, although at least weekly. The longer interval between the weight measurements was thought to be helpful to see trends in the data.

Daily it doesn’t change very much but weekly I think it does and therefore if you’ve done well you can see you’ve done well which motivates you to continue. (female)

I think I found the trends between weeks a bit easier to kind of mentally take something from than day to day. (female)

Some participants also preferred the weekly weighing as it allowed them to avoid judgement after an unhealthy day.

I think I would prefer to have this kind of longer window where I can say well okay like I ate too much cake yesterday or something so now […] I’ve got a couple of days where I can undo the damage. (male)

Overall, preferences for daily or less frequent weighing were dependent on how useful the daily weighing was perceived to be. This may explain why more men were willing to continue daily weighing than women (7/9 men vs. 4/15 women), since we found that men had fewer problems interpreting weight changes than did women.

## Discussion

### Principal results

The framework analysis revealed considerable differences in the extent to which self-regulation steps occur naturally after weighing. While participants compared their weight measurement to an expectation or goal and reflected on previous behaviour in the majority of cases, it was very rare that participants of either gender spoke of plans to take weight loss actions. Participants who frequently reflected on their behaviour were more likely to perform all other steps of the cognitive part of the self-regulation process. In exploratory regression analysis, specific action planning was the only self-regulation step that emerged as a significant predictor for weight loss.

In the thematic analysis, we found that weighing was an emotional topic for most participants, especially women. Weighing was perceived as useful for weight loss by the large majority of participants and helped participants realise that they had to make changes to their behaviour in order to lose weight. Some participants also reported that they felt the weighing influenced their behaviours throughout the day, helping them to remain more disciplined.

Difficulties with interpreting weight measurements emerged as a barrier to self-regulation. While for some participants a clear cause and effect relationship of their behaviour and weight was apparent, for others, mostly female participants, the daily fluctuations made it difficult to see the direct impact of their actions and impeded their ability to keep an overview of data trends. When participants were unable to make proper use of the daily feedback, they were more likely to want to continue weighing less frequently.

### The role of the cognitive self-regulation steps

Our results provide the first evidence that the comparison and reflection processes of the self-regulation process occur naturally after weighing in the majority of cases. Based on our occurrence and correlation findings, our participants seemed to follow the order of the self-regulation process, although not everyone reached the final stages, as evidenced by decreasing occurrence rates. Reflection on behaviour might be an important intermediate step between comparison and action planning, as it was significantly correlated with both, and comparison was not directly related to action planning.

Specific action planning emerged as the only significant predictor of weight loss, indicating that this step of the self-regulation process is the key contributor to weight loss effects found in previous studies. This finding aligns well with the literature on implementation intentions. Implementation intentions are similar to specific action planning in that they also specify how, when, and under which circumstances an action is to be performed (Hagger & Luszczynska, 2014). A randomised controlled trial by Luszczynska and colleagues showed that adding implementation intentions for diet and exercise behaviours to a weight loss programme significantly increased weight loss success (Luszczynska, Sobczyk, & Abraham, 2007). Similarly, a study by Benyamini et al. found that adding an action planning intervention to a weight loss programme elicited significant additional weight loss (Benyamini et al., 2013). Support for the importance of the specificity of action plans comes from a study aiming to increase exercise, which found that the specificity, rather than quantity of implementation intentions predicted physical activity outcomes (de Vet et al., 2011). In a meta-analysis of 94 studies investigating the impact of implementation intentions on behaviour performance across a vast range of behaviours, including health behaviours, a significantly positive medium-to-large effect was found (Gollwitzer & Sheeran, 2006). Another review concluded that defining if-then plans, making contingency plans, and incorporating relevant cues for action in the plan play an important role in the success of this strategy (Hagger & Luszczynska, 2014).

Specific action planning only occurred very rarely in our study. Similarly, de Vet et al. (2011) found that even when prompting participants to be more specific in their action plans, less than 10% of participants specified the context of all their action plans, and more than 60% did not even form a single specific action plan. This matches the conclusion of Hagger and Luszczynska (2014) who recommended that researchers should guide participants in forming implementation intentions in order to increase chances that they are formed correctly. Hence, the evidence suggests that participants need support in performing this last step of the self-regulation process, but that successful implementation might be a simple, yet effective, approach to weight loss.

### Experiences with daily weighing

Our qualitative analysis showed that weighing elicited emotional reactions. Similarly, a qualitative study by Zheng and colleagues, investigating the experiences with self-weighing in a weight loss trial, also found that participants reported feelings of frustration when expected weight loss did not materialise, and positive emotions when weight measurements were lower than expected (Zheng et al., 2018). Some of our participants found the intensity of their emotional reaction excessive. A positive finding of our study was that nine participants reported habituating to weighing, making it a less emotional and stressful task after a while. Similarly, a recent paper examining self-weighing perceptions over the course of a 12-month long weight loss trial reported that participants found weighing more positive and less frustrating by the end of the study (Fahey, Klesges, Kocak, Wayne Talcott, & Krukowski, 2018). Hence, a simple approach to making weighing a better and less emotionally upsetting experience might be to introduce regular self-weighing for an extended period of time in order to help people habituate to the process.

The overwhelming majority of participants in our study reported that they perceived regular weighing as a useful tool for weight management. Many found that weighing provided them with strong feedback about how their behaviour needed to change in order to lose weight. Some stated that they had been in denial about their weight loss attempts. Similarly, participants in the Zheng et al. (2018) study reported using self-weighing as a feedback tool for evaluating the effectiveness of their actions, allowing them to adjust their behaviour to better suit their goals.

Some participants in the present study stated that weighing helped them stay disciplined and make more healthy choices throughout the day. Hence, even though participants only rarely engaged in action planning, their behaviour was still influenced by the weighing to some extent. This corresponds to findings by Zheng et al. (2018), whose participants stated that the prospect of weight loss and observing weight loss motivated them to continue with weight loss behaviours. Similarly, studies from a laboratory setting have shown that weighing oneself significantly reduces subsequent snack consumption (Gupta, Wang, Corona, & Levitsky, 2017).

Although all participants wanted to continue weighing themselves regularly, and hence saw some value in checking in with the scales, the interpretation of daily fluctuations was a barrier to the constructive use of weight measurements. Many participants felt that the daily weight changes did not always reflect previous behaviours and that timing of the weighing, as well as food and drink intake, influenced the weight measurement. This is similar to the findings of Zheng et al. (2018), whose participants also realised that other factors than body fat gain or loss influenced their daily weight change. In our study, the mismatch between expectations and reality led to feelings of frustration and helplessness in some participants. Further work is needed to explore ways in which to best address this. However, participant responses highlighted helpful components of daily self-weighing too, including how it became habitual and helped them keep on track with their goals. It might therefore make sense to separate daily weighing from self-regulation, such that participants weigh daily but are prompted to self-regulate only on a weekly basis based on the trend of the week’s data. Since the day-to-day fluctuations seemed to differ in magnitude across participants and hence impeded them to different extents, future studies might also want to test assigning participants to different weighing frequencies, depending on the magnitude of their day-to-day weight fluctuations.

Although our participants realised the importance of interpreting long-term trends rather than daily changes, participants struggled to keep an overview of their weight loss progress, making them want to weigh on a less regular basis. Weight-tracking might be an effective remedy to this problem. In the Zheng et al. (2018)’s trial, participants specifically stated that they found the weight-tracking component of the intervention very useful as it allowed them to get an overview of their weight loss progress. An analysis of user reviews of weight-tracking apps found that the visualization of progress and feedback on weight loss success provides motivation and keeps users on track with their goals (Frie et al., 2017). In the present study, fewer than half the participants tracked their weight, but nearly all stated that it would have been useful to do so. Most studies implementing self-weighing also include a self-monitoring method, such as a diary (Lally et al., 2008), a self-monitoring app (Carter, Burley, Nykjaer, & Cade, 2013) or a web-based tracking tool (Pacanowski & Levitsky, 2015; Steinberg et al., 2013). These trials may have therefore successfully supported their participants in interpreting trends in their weight data, which might explain partly why these interventions were effective for weight loss.

Another barrier to self-regulation emerging from our analysis was related to unrealistic expectations. Our participants expected to see drastic weight changes after a day of vigilance and similarly expected their weight to increase sharply after a ‘cheat day’. Unrealistic expectations have previously been reported in the context of overall weight loss programmes (Foster, Wadden, Vogt, & Brewer, 1997; Linde, Jeffery, Finch, Ng, & Rothman, 2004; Wadden et al., 2003). Here, we show that unrealistic expectations also occur on a day-to-day basis. Although the impact of unrealistic expectations is contested, with some studies indicating that it does not affect weight loss outcomes (Linde et al., 2004) or even that weight loss outcomes are better for unrealistic goal-setters (Linde, Jeffery, Levy, Pronk, & Boyle, 2005), there is some evidence suggesting that unrealistic expectations may cause attrition from weight loss programmes (Dalle Grave et al., 2015; Dalle Grave et al., 2005; Moroshko, Brennan, & O'Brien, 2011). It is conceivable that continuous regular self-weighing would help participants adjust their daily expectations to more realistic values but further research is needed to explore this possibility.

### Strengths and limitations

We had strong engagement from participants in this study, and hence our data are not much affected by attrition bias. Sending reminders every other day and calling participants every two weeks allowed us to stay in touch and respond to problems. The results of this study are thus based on 498 think-aloud recordings, which were captured in the moment of weighing and provide unfiltered information on participants’ thoughts and feelings. This is the first study to have examined thought processes during and right after weighing in this detailed way. Compared to interview studies, our think-aloud method may be less affected by questions or prompts from the researcher. The information gathered from the think-aloud recordings was further supported by elaborations from the participants in over 700 minutes of follow-up interviews. This allowed us to gather reflections on self-weighing and put the think-aloud data in a broader context without influencing the experience of weighing itself.

One limitation is that it is likely that participation in the study acted as a low intensity intervention (Pacanowski, Bertz, & Levitsky, 2014). Some participants commented that the knowledge of participating in a research study motivated them to weigh every day and stay disciplined and committed to their goal. Previous research has shown that even control groups receiving no intervention but being followed up lose weight during clinical trials (Johns, Hartmann-Boyce, Jebb, Aveyard, & Behavioural Weight Management Review Group, 2016). The think-aloud task might have influenced participants’ self-regulation response to weighing. Sixteen participants reported that they felt thinking aloud led to more reflection on the weight outcome than would have naturally occurred. Hence, the occurrence of self-regulation in this study might be an over-estimation of reality, but it is unlikely that thinking aloud distorted the balance between the different self-regulation steps.

Since this study focussed on the cognitive processes following weighing, we cannot make conclusions about the day-to-day performance of weight loss actions. However, the connection between intentions and behaviour has been studied extensively elsewhere (Sheeran and Webb, 2016).

Even though all of our participants were trying to lose weight during the study, only a few of them were successful. It is conceivable that self-regulation occurrences and weighing experiences would have been different in a population which successfully lost weight. Further research is needed to investigate this possibility.

Another limitation is that our sample was small and not representative of the general population in that 22 participants had a university degree or equivalent. This limits the generalizability of our results as education can be viewed as a marker of socioeconomic status, and research has found that lower socioeconomic status is associated with lower levels of executive functioning (Marteau & Hall, 2013). One strength, however, is that our sample was relatively ethnically diverse. Due to the small sample size and the design of this study, the results of our exploratory regression analysis, showing an effect for specific action planning, need further investigation as well.

We quantified some of the data collected in the semistructured interviews. In hindsight, it might have been better to have collected this data in a quantitative way, for example through a questionnaire. However, the interviews allowed us further insights and supported the themes we identified during the inductive thematic analysis of the think-aloud recordings, thus strengthening our qualitative analysis.

## Conclusion

In summary, this study advances the field of self-regulation research by providing the first data on the cognitive processes following self-weighing. The results show that few people complete the self-regulatory process. Specific action planning is implemented particularly rarely, despite being a predictor of weight loss success. We also demonstrated that there are key barriers to self-weighing and self-regulation. These included difficulties interpreting weight changes due to unaccountable fluctuations, problems with remembering previous readings, and unrealistic weight loss expectations. In our discussion we addressed potential approaches to these barriers, which may improve the design of interventions that utilise self-monitoring and self-regulation components. Despite reported issues with daily weighing, our participants appreciated the learning that self-weighing engendered and intended to continue doing it.
